# Retraction: Carboxyamidotriazole-Orotate Inhibits the Growth of Imatinib-Resistant Chronic Myeloid Leukaemia Cells and Modulates Exosomes-Stimulated Angiogenesis

**DOI:** 10.1371/journal.pone.0224237

**Published:** 2019-10-16

**Authors:** The PLOS ONE Editors

After publication of this article [1], concerns were raised about results reported in Figures 1 and 3:

- In Figure 1b, the p-Crkl panels for LAMA84R and K562R appear similar.
- In the LAMA84R Bcr-Abl panel of Figure 1b, lanes 1 and 2 appear similar.
- In the LAMA84R β- actin panel of Figure 1b, lanes 1, 5, and 7 appear similar, and lanes 2, 4, and 6 appear similar.
- Figure 3a in [1] appears similar to Figure 1c in [2].

The authors clarified that the duplication of the panel from [2] was due to an error in preparing Figure 3a for the *PLOS ONE* article.

They further noted that the original data supporting the results in Figure 1b are no longer available. They provided replication data in support of the Figure 1b results but without the original image data, we cannot resolve the concerns about the published figure. Furthermore, the replication results raised questions about the reliability of reported results pertaining to dose-dependent inhibition of total and phosphorylated Bcr-Abl and the sensitivity of CrkL phosphorylation to Carboxyamidotriazole-orotate (CTO) treatment.

In light of the unresolved concerns about Figure 1b, the *PLOS ONE* Editors retract this article.

All authors disagreed with retraction.

Figure 3a in [1] reports material from [2], (© 2011 UICC), and so this figure panel is excluded from the *PLOS ONE* article’s [1] CC-BY license. At the time of retraction, the article [1] was republished to note this exclusion in the Figure 3 legend and the article’s copyright statement.

## References

[1] Corrado C, Flugy AM, Taverna S, Raimondo S, Guggino G, Karmali R, et al (2012) Carboxyamidotriazole-Orotate Inhibits the Growth of Imatinib-Resistant Chronic Myeloid Leukaemia Cells and Modulates Exosomes-Stimulated Angiogenesis. PLoS ONE 7(8): e42310 10.1371/journal.pone.0042310 22879938 PMC3411738

[2] Taverna S, Flugy A, Saieva L, Kohn EC, Santoro A, Meraviglia S, De Leo G, Alessandro R. Role of exosomes released by chronic myelogenous leukemia cells in angiogenesis. Int J Cancer. 2012 5 1;130(9):2033–43. 10.1002/ijc.26217 21630268 PMC3236253

